# Diagnosis of an occult gastric adenocarcinoma by oral manifestations (acanthosis nigricans): A case report

**DOI:** 10.22088/cjim.12.0.383

**Published:** 2021

**Authors:** Pegah Mosannen Mozafari, Roham Salek, Ava Taghizadeh, Mohammad Javad Yazdanpanah, Hooman Mosannen Mozaffari, Elham Esmaeili

**Affiliations:** 1 Department of Oral Medicine, School of Dentistry, Oral and maxillofacial Disease Research Center, Mashhad University of Medical Sciences, Mashhad, Iran; 2 Cancer Research Centre Faculty of Medicine Mashhad University of Medical Sciences, Mashhad, Iran; 3 Department of Oral Medicine, School of Dentistry, Birjand University of Medical Sciences, Birjand, Iran; 4 Cutaneous Leishmaniasis Research Center, Mashhad University of Medical Sciences, Mashhad, Iran; 5 Department of Gastroenterology and Hepatology, Mashhad University of Medical Sciences, Mashhad, Iran

**Keywords:** Myxosarcoma, Transthoracic Echocardiogram (TTE)

## Abstract

**Background:**

Acanthosis nigricans (AN) is a condition with an important characteristics of symmetrical areas of thickened skin with grayish brown hyperpigmentation. The mucosa may show a papillomatous surface, with or without hyperpigmentation. Lips and sites at risk of trauma may be affected and palmoplantar keratosis might also be present. In some rare cases, acanthosis nigricans presents as a sign of internal neoplasia, mostly a gastrointestinal cancer, and is called malignant acanthosis nigricans (MAN).

**Case Presentation:**

In this study, a 55-year-old female Iranian patient with malignant acanthosis nigricans (MAN) is reported. She was seeking esthetic treatment for her oral and perioral regions. The peculiarity of this case is simultaneous skin manifestation consistent with MAN, “tripe palms” (TP) and Leser-Trélat (LT) sign and mucosal changes in the oral cavity such as papillomatosis and roughened surfaces of the lips, hard palate and buccal mucosa. These changes harbored gastric adenocarcinoma stage T3 N3, but the patient was asymptomatic except for pruritis.

**Conclusion:**

There is an urgent need to suspect a correlation between oral and skin changes and the possibility of an internal neoplasia, therefore it is of utmost importance to refer these patients for early diagnosis of the underlying disease. This would improve the prognosis and lessen the consequences to a great extent.

Some cutaneous and mucosal changes should be looked into as important signs leading to the diagnosis of systemic diseases. In case of a malignancy, early detection of these changes results in early diagnosis of neoplasia, and therefore, an improved prognosis (1, 2). Acanthosis nigricans (AN) is a condition with important characteristics as follows: symmetrical areas of thickened skin with grayish brown hyperpigmentation, along with a velvety surface that might become verrucous and present as papular lesions (acrochorda).

 It involves skin folds, mainly the neck and the antecubital and popliteal fossa. The mucosa may show a papillomatous surface, with or without hyperpigmentation.

 Lips and sites at risk of trauma may be affected and palmoplantar keratosis might also be present. Palms may sometimes show tylosis or a more evident keratosis with a wrinkled aspect which is known as “tripe palms,”(TP) and is associated with an underlying neoplasia in 94% of cases.  

Generalized pruritus is a common complaint, and alopecia has also been reported. In some rare cases, acanthosis nigricans presents as a sign of internal neoplasia, mostly a gastrointestinal cancer, and is called malignant acanthosis nigricans (MAN) (1, 2). The Leser-Trélat (LT) sign, which involves multiple seborrheic keratoses with an abrupt onset together with widespread skin papillomatosis, can occur simultaneously as MAN. The most prominent malignancy associated with acanthosis nigricans is intra bdominal adenocarcinoma, with 45% of the cases accounting for gastric adenocarcinoma. Other carcinomas said to be associated with acanthosis nigricans are carcinomas of the lung, liver, uterus, breast, and ovaries, along with lymphomas and mycosis fungoides (2, 3). 

The majority of AN cases appear simultaneously with the tumor (61.3%), but AN can present prior to or after tumor development by 17.6% and 21.1% respectively. The neoplasia is believed to produce cytokines, such as transforming growth factor α (TGF-α), stimulating the proliferation of keratinocytes, hence, cutaneous lesions are highly likely to be resolved followed by tumor resection. Other treatment options such as acitretin, oral isotretinoin tretinoin, and topic ammonium lactate are also suggested (1, 3).

TP, which also goes by the term “acanthosis palmaris” and “AN of the palms”, is the result of an accentuation of the dermatoglyphic lines, causing a wrinkly, grooved appearance on the palmar skin. The plantar region can also be affected. Tripe palms is associated with a form of malignancy in 90% of the cases. Mostly, it coexists with AN and less commonly with the LT sign, suggesting that there may be mutual alterations involved. TGF-α may be a common factor directing the pathogenesis of tripe palms as well as AN. Both conditions have parallel evolution to cancers and both show similar histopathologic changes. The occurrence of solitary tripe palms without the presentation of AN could lead us to suspect the possibility of pulmonary squamous cell carcinoma (1, 3).

In this study, a 55-year-old female Iranian patient with MAN is reported. The peculiarity of this case is simultaneous skin manifestation consistent with MAN, TP, and LT sign and mucosal changes in the oral cavity; these cutaneous preneoplasia harbored gastric adenocarcinoma stage T3 N3, but the patient was asymptomatic except for pruritis. Since AN has been associated with malignancy, recognizing MAN earlier may improve with the treatment of the underlying malignancy.

## Case presentation

A 55-year-old obese woman attended an appointment in the Oral Medicine Department of Mashhad Dental School with the sole complaint of gradual blackening of perioral region, deformation of the lips. She was seeking esthetic treatment for these changes. She mentioned the same type of blackening in body skin folds, axilla, groin, shoulders and back of the neck and between the breasts with associated itching. She recalled the onset of skin changes on her forehead and chin going back to six months before her admission. The patient revealed that all the skin changes were associated with itching sensation three months after their initial appearance. The patient’s previous assessments had failed to establish a definite diagnosis. erythrocyte sedimentation rate :24 mm/h (normal range:0-20), cholesterol 216 (medium risk:200-240) ,CRP: positive, ANA: 15 (<10 :negative ,>10 positive ). No pathology was observed in the sonograms of the uterus, ovaries and adrenal glands. Pap smear results were also normal. The abdomino-pelvic ultrasonography reported stomach wall thickening and infiltration in the distal gastric antrum. In addition, several pathologic adenopathies (15 to 25 mm ) were observed in the celiac region, which were attributed to lymphoma or another malignant neoplastic lesion. Due to sonographic findings, endoscopic examination was recommended. In esophago-gastro duodenoscopy, diffused infiltration was seen in the body and the proximal part of the antrum.

 The biopsy depicted gastric adenocarcinoma.The patient was referred to the oncologist and underwent preoperative chemotherapy followed by surgery and postoperative chemoradiotherapy. All the treatments were well-tolerated by the patient with the only exception being an allergic reaction to a chemotherapy drug called oxaliplatin during the third cycle of preoperative chemotherapy. After a 12-month follow-up since the final diagnosis, the patient is now in good condition and without any signs and symptoms of relapse. On physical examination the black and velvety skin changes are faded and there was not any sign of mucosal and lip pappillomatosis. 

**Fig 1:**
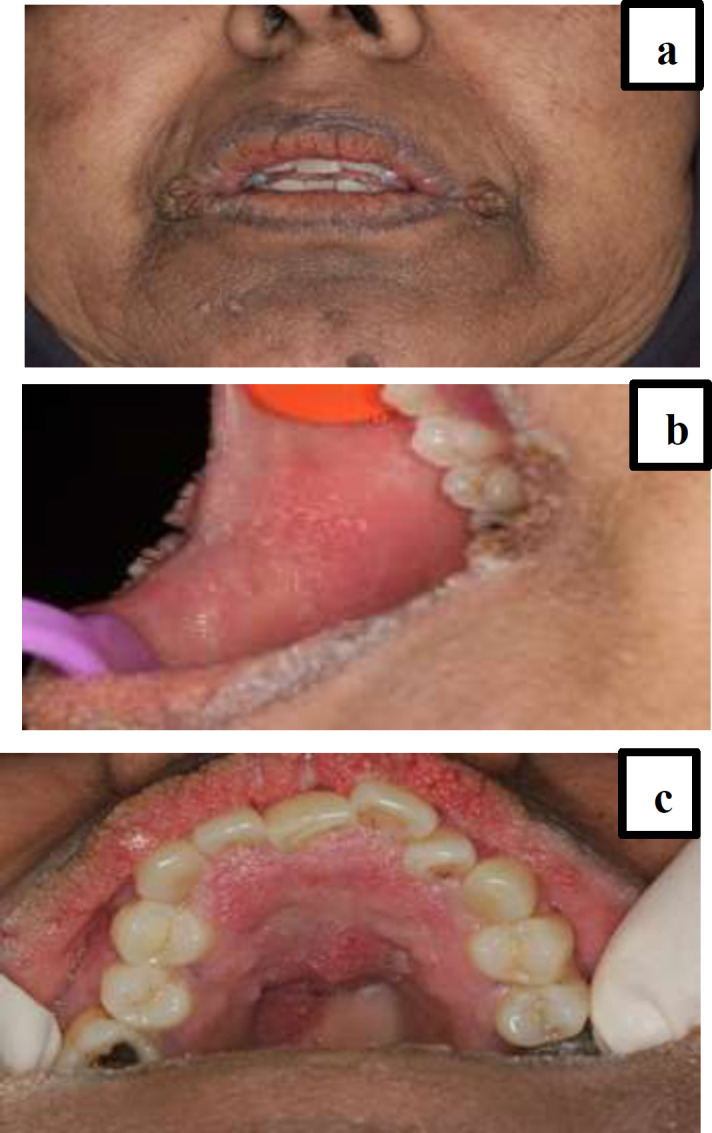
A: Papillomatosis with darkening of perioral

B:Papillomatosis on buccal mucosa. C:Enlargement of the mucosa in the hard palate with a papillomatous appearance.

**Fig 2:**
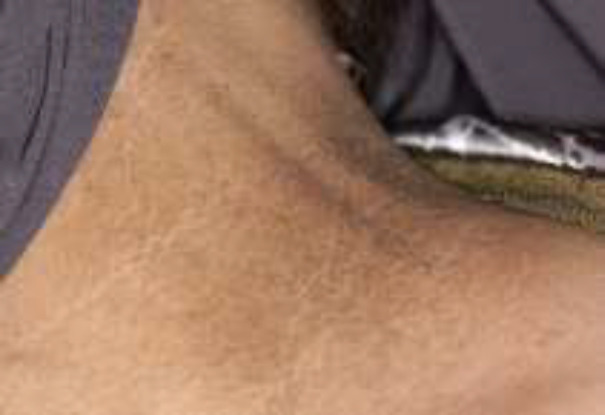
Pigmentation on neck

**Fig 3:**
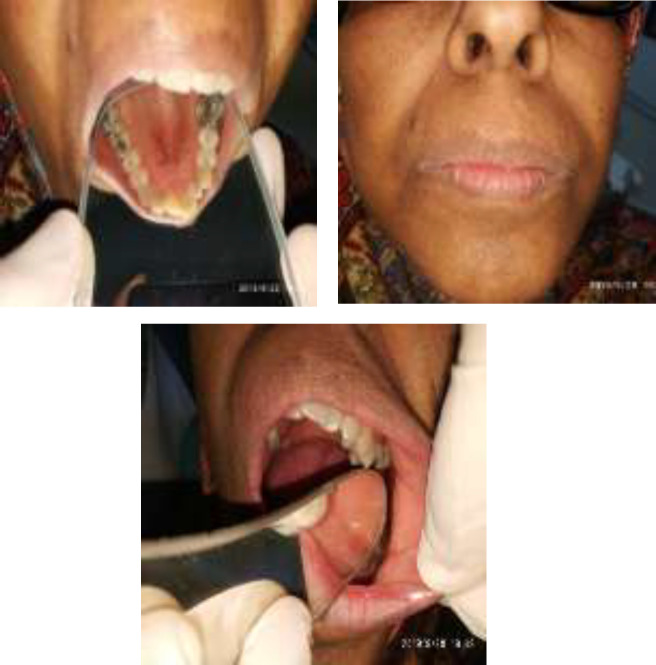
Fallow up 1 year after and remission of papillomatosis and pigmentation appearance

## Discussion

AN is a condition which is diagnosed through characteristics such as hyperkeratotic symmetrical discoloration along with verrucous lesions. The major sites of involvement are the axilla, intermammary area, navel, antecubital region and also around the anus. There are many classifications based on the clinical appearance: benign, obesity-associated, syndromic, unilateral, acral, drug-induced, malignancy-associated and mixed type. In general, MAN is not as common as AN and is not triggered by cancer. However, the significance of diagnosing MAN lies within the fact that it could be seen as a cutaneous marker for predicting an internal malignancy (4). TP which is the hyperkeratosis of the palms with a wrinkly appearance, has been linked to internal malignancies more often. In a research done by Cohen at al, it was reported that over 90% of cases with TP had an internal malignancy. What more, in the same study, it was found that AN and LT were also associated with TP by 70% and 10% respectively (4, 5). What is interesting about these associations and about this case in particular is that cutaneous manifestations of MAN and LT and TP only very rarely coexist in one single individual. There have been only two cases reported on Pubmed with all three conditions coexisting.

In the present case report, an obese Iranian woman with a medical history of controlled systemic lupus erythematosus and hypothyroidism and a drug history of levothyroxine and prednisolone was admitted to the oral medicine department of Mashhad Dentistry School. A prior biopsy from the lip lesions and the perioral skin was done in the ENT ward. The biopsy report indicated cheilitis and papillomatous reaction. The patient was advised to seek esthetic treatment. The patient sought esthetic treatment for removal of lip bumps and blackening of the perioral region. 

She had oral, perioral and skin lesions consistent with MAN. She also had LT and TP based on clinical findings. The patient had no other symptoms to suggest a malignancy. However, she was referred to an internal medicine specialist and laboratory tests were requested as well as multiple sonograms. A final endoscopic exam followed by a biopsy confirmed our suspicion of malignancy and gastric adenocarcinoma was confirmed. The patient was thus referred to an oncologist and underwent treatment. The patient is currently in a stable condition and without any signs and symptoms of relapse, after being followed for a year since the final diagnosis was made. On physical examination, the black and velvety skin changes are gradually fading and there was not any sign of mucosal and lip pappillomatosis.

It is important to bear in mind that the clinical manifestations of MAN will resolve once the malignancy has been suppressed or the tumor removed. A significant issue is when to become more suspicious of a malignancy with a patient showing clinical symptoms of AN. As a general rule, it is extremely logical to screen patients for internal malignancies once they have a sudden manifestation of AN while being over 40 years of age. This becomes more necessary to investigate when the individual is free of previous endocrine diseases and symptoms and is presenting LT and TP signs as well. The patient also needs to undergo hematological work-ups as well as liver and renal function tests, chest x-ray, bronchoscopy, abdomen CT and ultrasound and possibly an oesophagoduodenoscopy to rule out any internal neoplasia. Muhammad Rizvan et al. has reported a recent case of MAN in 2019, of 62-year-old man with generalized itching, darkening of skin, velvety verrucous plaque on oral mucosa, hyperkeratotic soles and a positive tripe palm. The patient was suspected of having an internal malignancy and was referred for further investigations. The final diagnosis was an undifferentiated malignant hepatic neoplasm (5).

In another study done by Han-Wen Chu et al. in 2019, a 59-year-old woman was admitted with clinical presentations of lip papillomatosis and axillary area skin hyperkeratosis and hyperpigmentation.The patient’s final diagnosis was MAN related to metastasis of endometrial adenocarcinoma with clinical stage-IVB according to the International Federation of Gynecology and Obstetrics. This case is similar to ours that the perioral and skin presentations are associated with an internal malignancy. However, unfortunately this patient passed away after a four-month cancer treatment period (6).

In a case report done by Lindsey et al. in 2018, a 78-year-old was admitted to the department of dermatology with a systemic history of hypothyroidism, hyperlipidemia and type 1 diabetes. The patient had pruritis and morbilliform rash extending from her chest and abdomen to proximal extremities from a month ago. What more, she had developed lichenified plaques in the aforementioned regions for which she had received therapy with topical Tacrolimus but had not healed. The patient was biopsied from her plaque which showed a non-specific dermal inflammation. The patient presented with new hyperkeratotic rugated plaques on her chest and neck two weeks after her biopsy, consistent with AN. At the same time, stuck-on waxy papules were also evident in her neck and chest, correlating with seborrheic keratosis seen in LT. Unlike our patient, this case had no oral mucosal involvement. The patient’s additional symptoms were weight loss, night sweats, fever and chill. Upon referral to the gynecology oncology department, a stage 3C serous fallopian tube carcinoma and a synchronous low-grade endometrioid adenocarcinoma of the endometrium was diagnosed. The LT sign might be associated with fallopian tube carcinoma (7). 

Deen et al. reported a case in 2017 of a 28-year-old obese woman who was referred to the dermatology clinic with an 8-year history of hyperpigmentation on her face presenting as an eruption, and also present in the neck, axillae and forearms area, which had increased in the amount of pigmentation in the past year. The patient had been using topical corticosteroids for the past 5 years which did not have much healing effect. The patient’s history revealed irregularity of menstrual cycles along with menorrhagia. The patient also had skin thickening in the axillae, arms, abdominal folds and ears. Macrocephaly, a prominent nose and a buffalo hump with a round face were additional findings. The patient had no oral presentations. Punch biopsies from her skin lesions confirmed a diagnosis of AN. Upon diagnosis, other systemic work-ups and investigations were carried out to exclude neoplastic lesions. The only abnormal findings were extremely low FSH and LH and an elevation in testosterone and free androgen index. This led to a referral for hysteroscopy with dilation and curettage which when examined histopathologically, confirmed the diagnosis of a grade 1 adenocarcinoma of the endometrium (8).

In 2015, Zhang et al. reported a case of a 41-year-old female patient with a one-year history of blackening of body folds and creases which were initially evident in her axillae and groins but spread across her neck, elbows umbilicus and anus shortly as keratotic plaques with velvety surface. She also mentioned a wart-like lesion with pruritis on her arm dating to six months prior. The overall state of the patient was good, with no additional symptoms. Unlike our case, there were no oral findings. Skin biopsy from axilla confirmed the diagnosis of AN, while biopsy from the warty lesion confirmed the diagnosis of keratosis seborrheica. Further investigation was carried out and a gastroscopy showed stiffness, peristaltic difference and erosion. The histopathological findings confirmed gastric adenocarcinoma (9). Another case report by Singh and Rai in 2013, reported a 47-year-old woman presenting with a 14-month progressive hyperpigmentation and hyperkeratosis spread throughout her whole body. The patient had a lower abdominal mass 8 months before admission and the mass had a hard consistency. There were systemic symptoms such as fatigue, weight loss and night sweat in the past 8 months. The patient’s laboratory findings showed an iron deficiency anemia, elevated levels of alkaline phosphatase and a reversed albumin to globulin ratio. The patient was referred for a definite diagnosis. The final diagnosis revealed MAN associated with ovarian malignancy (10). Our present case report together with all those aforementioned, highlight the fact that there is an urgent need to suspect a correlation between oral and skin changes and the possibility of an internal neoplasia, therefore it is of utmost importance to refer these patients for early diagnosis of the underlying disease. This would improve the prognosis and lessen the consequences to a great extent.
